# Complementary and Alternative Medicine and Cancer Survivorship

**DOI:** 10.1155/2012/850429

**Published:** 2012-12-19

**Authors:** Alyson L. Huntley, Beverley de Valois, Tieraona Low Dog, Francesca Borrelli

**Affiliations:** ^1^School of Social and Community Medicine, University of Bristol, Canynge Hall, Bristol BS8 2AP, UK; ^2^Lynda Jackson Macmillan Centre (LJMC), Mount Vernon Cancer Centre, Middlesex UB8 2DL, UK; ^3^Arizona Center for Integrative Medicine, University of Arizona, Tucson, AZ 85724-5153, USA; ^4^Department of Experimental Pharmacology, University of Naples Federico II, Naples 80131, Italy

In the UK, the Cancer Reform Strategy 2007 outlined the need for a National Cancer Survivorship Initiative to improve the care and support provided for those living with and beyond cancer. This is reflected across the world with health care research investigating new models of cancer care which include helping people to live healthily after cancer, helping them to prevent recurrence of cancer, to return to as normal a life as possible after treatment, and to support people living with active or advanced cancer.

The US National Cancer Institute defines an individual as a cancer survivor from the time of cancer diagnosis, through the balance of his or her life. Evidence from qualitative studies informs us that supportive care is wanted by cancer patients but it is felt there is a lack of appropriate support services. Complementary and alternative medicine (CAM) can play an important role in this approach.

This is why we felt it was important to propose a special issue on CAM and cancer survivorship. We also felt it was important to take a broad view not only across a wide range of therapies and interventions, some of which are unproven, but equally across a range of methodologies from in vitro studies to qualitative reports. We are pleased to say we achieved that with this special issue.

Four of the included papers discuss in vitro studies investigating the mechanisms of Traditional Chinese Medicine (TCM) and Ayurvedic medicine on cancer cell biology. Two reviews discuss the role of inflammation and its treatment in cancer. M. Sun and colleagues discuss Kushen, which has a long history of use in TCM to treat inflammatory diseases and cancer. This review consolidates the evidence base for Kushen in modulating molecular pathways in tumours. The review by V. N. Sumantran contains a discussion on the shared pathology of inflammation of cancer and metabolic syndrome in the context of the Ayurvedic concept of “Ama.” A further review by Z. Wang and colleagues discusses the current understanding of the potential mechanisms of TCM in cancer therapy, and specifically the cancer glycolytic pathway. We have also included a study led by S. Sheikh which provides evidence for an Ayurvedic herbomineral preparation and its effects on the human cancer cell lines, and apparent lack of toxicity both in animals and human subjects.

Nutritional and herbal therapies are also covered in four clinical papers. H. Othman writes on a novel approach to the role of honey in health care in developing countries. Whilst the author discusses honey as a natural immune booster, anti-inflammatory and antimicrobial agent, cancer “vaccine,” and a promoter for healing chronic ulcers and wounds, he also takes the argument further by suggesting that bee farming could provide both income and health benefits in developing countries.

M. C. S. Araujo controlled trial of *Uncaria tomentosa* shows that it may be effective in aiding recovery from chemotherapy-induced neutropenia for women with breast cancer. In a review of 1,217 case reports sourced from four Chinese databases (from 1958 to 2011), G. Yang et al. report that chemo-/radiotherapy-induced leukopenia was the most common type of condition treated by TCM. The paper by J. N. Lai and colleagues describes prescription patterns of TCM for breast cancer in Taiwan and shows that 81.5% of women with breast cancer use TCM and 18% of them seek TCM for treating their breast cancer. Commonly used herbals were Jia-wei-xiao-yao-san, dang qui, and ren shen, and the authors conclude that the effects of these herbs should be taken into account by healthcare providers.

Two papers focus on the development of tools for CAM research. Patient reported outcomes (PROs) used in yoga intervention studies for cancer survivors from 2004–2011 are explored by S. N. Culos-Reed. This research provides new directions for examining clinical significance using PROs such as quality of life and psychosocial or symptom measures. In addition, J. J. Mao et al. report on the development and validation of a measure of cancer survivors' attitudes and beliefs about CAM. This 15-item instrument is based on the theory of planned behaviour and has a 3-factor structure: expected benefits, perceived barriers, and subjective norms related to CAM use by cancer patients. This study provides preliminary evidence that the instrument produces reliable and valid scores to measure attitudes and beliefs related to CAM use among cancer patients.

This special issue covers several aspects of physical and mind body therapies in cancer care. J. L. Ryan and colleagues look at CAM interventions for cancer related stress including mindfulness, yoga, Tai Chi Chuan, acupuncture, energy-based techniques, and physical activity. Whilst the evidence base for these studies is often limited, the authors conclude that some these approaches could be integrated into standard cancer care.

A novel mixed method review of dragon boat racing for breast cancer survivors provides a fascinating insight into this supportive therapy. This narrative review summarizes findings from quantitative and qualitative research supporting the hypothesis that dragon boat paddling is safe for women recovering from breast cancer, and showing that it has been embraced as a complementary exercise therapy by cancer survivors.

We have two studies which examined the role of massage for cancer patients using both quantitative and qualitative methodologies. A pilot study by N. A. Hodgson et al. investigates the effects of reflexology and Swedish massage therapy on physiologic stress, pain, and mood in older cancer survivors residing in nursing homes. They showed a significant decline in salivary cortisol and pain, and improvements in mood. S. L. Ackerman and colleagues describe parent caregivers' experience of the effects of massage and acupressure for their children undergoing haematopoietic cell transplantation. Benefits for both children and parents are described.

This special issue demonstrates the breadth of CAM research for cancer survivorship, including many novel and innovative approaches. We hope you find it stimulating and useful.

*Alyson L. Huntley* *Alyson L. Huntley* *Beverley de Valois* *Beverley de Valois* *Tieraona Low Dog* *Tieraona Low Dog* *Francesca Borrelli* *Francesca Borrelli*

